# Alzheimer's disease - a neurospirochetosis. Analysis of the evidence following Koch's and Hill's criteria

**DOI:** 10.1186/1742-2094-8-90

**Published:** 2011-08-04

**Authors:** Judith Miklossy

**Affiliations:** 1 International Alzheimer Research Center, Prevention Alzheimer Foundation, Martigny-Combe, Switzerland

**Keywords:** Alzheimer's disease, bacteria, *Borrelia burgdorferi*, dementia, infection, Lyme disease, periodontal pathogen, spirochetes, *Treponema*, syphilis

## Abstract

It is established that chronic spirochetal infection can cause slowly progressive dementia, brain atrophy and amyloid deposition in late neurosyphilis. Recently it has been suggested that various types of spirochetes, in an analogous way to *Treponema pallidum, *could cause dementia and may be involved in the pathogenesis of Alzheimer's disease (AD). Here, we review all data available in the literature on the detection of spirochetes in AD and critically analyze the association and causal relationship between spirochetes and AD following established criteria of Koch and Hill. The results show a statistically significant association between spirochetes and AD (P = 1.5 × 10^-17^, OR = 20, 95% CI = 8-60, N = 247). When neutral techniques recognizing all types of spirochetes were used, or the highly prevalent periodontal pathogen Treponemas were analyzed, spirochetes were observed in the brain in more than 90% of AD cases. *Borrelia burgdorferi *was detected in the brain in 25.3% of AD cases analyzed and was 13 times more frequent in AD compared to controls. Periodontal pathogen Treponemas (*T. pectinovorum, T. amylovorum, T. lecithinolyticum, T. maltophilum, T. medium, T. socranskii*) and *Borrelia burgdorferi *were detected using species specific PCR and antibodies. Importantly, co-infection with several spirochetes occurs in AD. The pathological and biological hallmarks of AD were reproduced *in vitro* by exposure of mammalian cells to spirochetes. The analysis of reviewed data following Koch's and Hill's postulates shows a probable causal relationship between neurospirochetosis and AD. Persisting inflammation and amyloid deposition initiated and sustained by chronic spirochetal infection form together with the various hypotheses suggested to play a role in the pathogenesis of AD a comprehensive entity. As suggested by Hill, once the probability of a causal relationship is established prompt action is needed. Support and attention should be given to this field of AD research. Spirochetal infection occurs years or decades before the manifestation of dementia. As adequate antibiotic and anti-inflammatory therapies are available, as in syphilis, one might prevent and eradicate dementia.

## Introduction

The recognition that pathogens can produce slowly progressive chronic diseases has resulted in a new concept of infectious diseases. The pioneering work of Marshall and Warren has established that *Helicobacter pylori *(*H. pylori*) causes stomach ulcer [1]. Also the etiologic agent of Whipple's disease was revealed to be another bacterium, *Tropheryma whippeli*. Recent reports have documented that infectious agents also occur in atherosclerosis, cardio- and cerebrovascular disorders [2-10], diabetes mellitus [11-16], chronic lung [17-20] and inflammatory bowel diseases [1,21-25], and various neurological and neuropsychiatric disorders [26-31].

Nearly a century ago, Fischer, Alzheimer and their colleagues [32,33] discussed the possibility that microorganisms may play a role in the formation of senile plaques. Historic data indicate that the clinical and pathological hallmarks of syphilitic dementia in the atrophic form of general paresis, caused by chronic spirochetal infection, are similar to those of AD. There is an increasing amount of data that indicates that spirochetes are involved in the pathogenesis of AD. This review presents historic and new data related to the involvement of spirochetes in AD. The goal was to critically analyze the association and causality between spirochetes and AD, based on the substantial amount of data available and on established criteria of Koch [34,35] and Hill [36]

## Pathological hallmarks and pathogenesis of Alzheimer disease

AD is the most frequent cause of dementia and is characterized by a slowly progressive decline of cognition and memory. Alzheimer described the characteristic cortical senile plaques and neurofibrillary tangles in the brain of a 51-year-old woman who suffered from presenile dementia [37]. Recently, it was pointed out that the presenile form, with onset before age 65, is identical to the most common form of senile dementia [38,39]. Therefore today the term AD is used for the designation of both presenile and senile cases.

The pathological hallmarks of AD are progressive brain atrophy and the accumulation of cortical senile plaques and neurofibrillary tangles. A fibrillary amyloid substance is deposited in senile plaques, formed by the aggregation of the 4.2-kD amyloid beta peptide (Aβ). Aβ is derived by proteolytic cleavage from a transmembrane amyloid beta precursor protein (AβPP). Neurofibrillary tangles contain paired helical filaments (PHFs), and the major component of PHFs is the microtubule associated protein tau. Granulovacular degeneration is another characteristic alteration of neurons in AD.

The origins of Aβ deposition, neuronal tangle formation and granulovacuolar degeneration still remain unclear. Various hypotheses were proposed to explain the pathogenesis of AD [40,41]. Mutations in AβPP, presenilin 1 and presenilin 2 genes are implicated in inherited, early onset AD, but the frequency of familial cases is very low [42]. The epsilon 4 allele of apolipoprotein E (ApoE4) was revealed to be a risk factor for AD [43]. Polymorphisms of various genes, including numerous inflammatory genes [44] are associated with AD. The AlzGene database (http://www.alzgene.org) assembles and organizes the increasing number of AD related susceptibility genes [45]. It provides a comprehensive, unbiased and regularly updated data on genetic studies performed in AD, including meta-analyses for various polymorphisms related to AD.

The relationship between the two major biological markers of AD Aβ and hyperphosphorylated tau is not clear. That the soluble form of Aβ and tau strongly interact [46] and that AβPP is also expressed in neurofibrillary tangles [47] suggest that these apparently different pathologies are linked in AD.

The critical role of chronic inflammation in AD is now widely recognized. The important role of neuroinflammation and the importance of IL-1 signaling were first documented by McGeer, Rogers and Griffin [48-50]. Cellular and molecular components of the immune system reactions including the membrane attack complex (MAC, C5b-9) are associated with AD cortical lesions [51-54] and non-steroidal anti-inflammatory drugs (NSAIDs) reduce the risk of 55-80% for AD [55-57].

## The clinical and pathological hallmarks of AD are similar to those of the atrophic form of general paresis

Historic observations show that the clinical and pathological hallmarks of AD are similar to those occurring in the atrophic form of general paresis [58,59]. Noguchi and Moore [60] by showing the presence of *T. pallidum *in the cerebral cortex of patients with general paresis provided the conclusive evidence that *T. pallidum *is responsible for slowly progressive dementia, cortical atrophy and local amyloidosis in the atrophic form of this chronic bacterial infection.

This form of general paresis is characterized by a diffuse, predominantly frontotemporal cortical atrophy. The characteristic pathological features comprise severe neuronal loss, reactive microgliosis and astrocytosis. Spirochetes form plaque-like cortical masses or colonies [61,62]. Pacheco e Silva [61,62] by analyzing the brains of more than 60 patients with atrophic general paresis reported that the number of spirochetes and spirochetal plaques increased with the severity of cortical atrophy. The morphology and distribution of *T. pallidum *colonies are identical to those of senile plaques. Spirochetes are more numerous in the hippocampus and frontal cortex [61,62] and accumulate without accompanying lymphoplasmocytic infiltrates. Another characteristic feature of the atrophic form of general paresis is the accumulation in the brain of "paralytic iron" [63]. Neurofibrillary tangles and amyloid deposition both occur in dementia paralytica [59,64-66]. Recent analysis of archival brain material of clinically and pathologically confirmed general paretic cases revealed that the local amyloid deposit in general paresis, as in AD, consists of Aβ [67].

## Association of spirochetes and Alzheimer's disease

That dementia associated with cortical atrophy and microgliosis also occurs in late stages of Lyme disease [68-73] caused by *Borrelia burgdorferi *(*B. burgdorferi*), suggested that various types of spirochetes in an analogous way to *T. pallidum *might cause dementia and brain pathology similar to AD.

Various Borrelia and Treponema species from the family *Spirochaetaceae *are responsible for diverse human diseases. From 36 known Borrelia species 12 cause Lyme disease or other borreliosis, which is transmitted by the bite of infected ticks. Relapsing fever is caused by nearly 20 species of *Borrelia recurrentis *and is transmitted by ticks and lice [74,75]. Near 60 diverse Treponema species were identified in subgingival pockets in human periodontal diseases [76,77]. These periodontal pathogen spirochetes comprise *Treponema denticola, Treponema socranskii, Treponema pectinovorum, Treponema amylovorum, Treponema lecithinolyticum, Treponema maltophilum and Treponema medium. Treponema vincentii *causes necrotizing fusospirochetal disease called Vincent angina. Many other Treponema species are present in the human genital mucosa. From the family *Brachyspiraceae*, two species of the genus *Brachyspira *(*B.*), *i.e. B. aalborgi *and *B. *(*Serpulina*) *pilosicoli *are responsible for human intestinal spirochetosis [78,79]. Spirochetes of the genus *Leptospira, *family *Leptospiraceae, *cause human leptospirosis.

### Detection of all types of spirochetes

To verify the hypothesis that several types of spirochetes may be involved in AD, 147 AD cases and 37 controls were analyzed using neutral techniques, which recognize all types of spirochetes.

In an initial study, helically shaped microorganisms were observed in 14 AD cases in the cerebrospinal fluid (CSF), blood and cerebral cortex [70]. They were isolated from the cerebral cortex, and cultivated from the blood in a modified Noguchi medium, which enables the cultivation of anaerobic spirochetes. They were absent in age-matched controls, which were without any AD-type cortical changes [70]. In three AD cases, spirochetes were also cultivated from the cerebral cortex in a synthetic Barbour-Stoenner-Kelly II (BSK II) medium [70]. Further scanning electron microscopy and atomic force microscopy analyses defined that these helically shaped microorganisms possess endoflagella and taxonomically belong to the order *Spirochaetales *[80]. Spirochetes were detected in the brains of 8 AD patients derived from another laboratory and in the blood of 5 living patients with AD-type dementia [81]. In addition to dark field, atomic force, electron and immune-electron microscopy analyses, immunohistochemical detection of spirochetes was also performed using spirochete and bacterial peptidoglycan (PGN) specific antibodies, and by using the nonspecific DNA marker 4',6-Diamidine-2'-phenylindole dihydrochloride (DAPI) and species-specific DNA as revealed by *in situ *hybridization (ISH) [70,80-86]. PGN is the building block of the cell wall of virtually all Eubacteria, including spirochetes, however, Mycoplasma and Chlamydia, which lack bacterial cell wall, do not show detectable PGN [87,88]. The morphology of helically shaped microorganism detected by spirochete or PGN specific antibodies is identical [compare Fig. seven G and H of reference 89]. PGN-immunoreactive helically shaped spirochetes were detected in the brains in 32 definite AD cases and in 12 cases with mild or moderate AD-type cortical changes [87,88]. Spirochetes were observed in senile plaques, neurofibrillary tangles, curly fibers and in the wall of cortical or leptomeningeal arteries exhibiting amyloid deposits [70,80-82]. Spirochete and PGN specific antigens were co-localized with Aβ [83,85]. Control brains without AD-type cortical changes were negative [70,83-85]. These observations have suggested that various types of spirochetes of the order *Spirochaetales*, might cause dementia and contribute to the pathogenesis of AD.

McLaughlin et al., [90] did not find spirochetes by dark field and electron microscopy in the brains of 7 AD cases tested. They observed spirochetes in the blood in one of 22 clinically diagnosed AD patients (Table 1). The spirochete illustrated by the authors corresponds to a regularly spiral vegetative form. It is not clear, whether the atypical, pleomorphic spirochete forms, which are common in blood and in infected tissues [89,91-93] were considered or not in this study. The authors have suggested that the spirochete observed could correspond to oral Treponema.

**Table 1:** Detection of spirochetes in Alzheimer disease

Authors	N	Mat	Meth	AD	CTRL	Cult	Serol
***Detection of all types of spirochetes***							

Miklossy, 1993 & Miklossy et al., 1994 [70,80]	27	Brain, Bl, CSF	DF, HC, IHC, EM, AFM	14/14	0/13**	14/14 (Bl 4/5)	Nd

Miklossy, 1994 [81]	12 5	Brain Bl	DF DF	8/8 5/5	0/4**	8/8 Nd	Nd Nd

Miklossy et al., 1995 [82]	24 10***	Brain	DNA-DAPI	20/20 10/10***	0/4**	Nd	Nd

Miklossy et al., 1996, Miklossy, 1998 [83,84]	54	Brain	IHC	32/32 12/12*	0/10**	Nd Nd	Nd Nd

McLaughlin et al., 1999 [90]	7 28	Brain Bl	DF, EM	0/7 1/22	0/6	Nd Nd	Nd Nd
							

**Total: Various types of spirochetes detected using neutral techniques**							
**Brain: AD N = 102, AD 64/71, CTRL 0/31", P = 4.8 × 10^-18^, OR" = 274, 95% CI = 32-11345**							
**Brain: AD, mild AD N = 114, AD 76/83, CTRL 0/31", P = 1 × 10^-19^, OR" = 325, 95% CI = 38-13440**							
**Brain, Bl, CSF: AD, mild AD N = 147, AD 82/110, CTRL 0/37", P = 1.1 × 10^-15^, OR" = 105, 95% CI = 13-4329**							
							

***Periodontal pathogen spirochetes***							

Riviere et al, 2002 [96]	34	Brain,	PCR, IHC	15/16	6/18	Nd	Nd

**Total: Periodontal pathogen spirochetes detected in the brain**							
**Brain: AD, N = 34, AD 15/16, CTRL 6/18, P = 3.6 × 10^-4^, OR = 30, 95%CI = 2.8-1364**							

***Borrelia burgdorferi***							

MacDonald & Miranda1987 [98]	2	Brain	IHC	1/1	0/1	+	Nd

MacDonald, 1988 [99]	1	Brain	DF, IHC	1/1		+	Nd

Pappolla et al., 1989 [103]	10	Brain	EM, IHC, Wbl	0/6	0/4	-	Nd

Miklossy, 1993 and Miklossy et al., 2004 [70,85]	27***	Brain Bl, CSF	Cult, IHC, EM, ISH, 16SrRNA	3/14***	0/13***	3/14	2/14

Miklossy, 1993 [70]	1	Brain	IHC	1/1		Nd	1/1

Gutacker et al., 1998 [104]	10	Brain	PCR	0/10		-	Nd

Marques et al., 2000 [105]	30	Brain	PCR	0/15	0/15	Nd	Nd

Riviere et al., 2002 [96]	34***	Brain	PCR, seq	5/16***	1/18***	Nd	Nd

Meer-Scherrer et al., 2006 [100]	1	Brain	PCR	1/1		Nd	1/1

MacDonald, 2006 [101,102]	11	Brain	PCR, IHC	7/10	0/1	Nd	1/1

**Total: All studies detecting *Borrelia burgdorferi***							
**Brain: AD N = 127, AD 19/75, CTRL 1/52, P = 2.9 × 10^-4^, OR = 17, 95%CI = 2-732**							

Total: All studies detecting spirochetes

Brain: AD N = 202, AD 90/131, CTRL 6/71, P = 1.7 × 10^-17^, OR = 23, 95%CI = 9-71

Brain: AD, mild AD N = 214, AD 102/143, CTRL 6/71, P = 1.5 × 10^-19^, OR = 26, 95% CI = 10-80

Brain, Bl, CSF: AD, mild AD N = 247, AD 108/170, CTRL 6/77, P = 1.5 × 10^-17^, OR = 20, 95% CI = 8-60

Data reviewed in the literature with respect to the detection of all types of spirochetes using neutral techniques and the specific detection of periodontal pathogen Treponemas and *Borrelia burgdorferi*. Results of the statistical analysis are given for each group and for all studies together. N = total number of cases investigated, AD = Alzheimer disease, CTRL = control, Mat = material, Meth = methods, Cult = culture, Serol = serology, AD = Number of AD cases positive for spirochetes/number of AD cases analyzed, CTRL = Number of control cases positive for spirochetes/number of control cases analyzed, Bl = blood, CSF = cerebrospinal fluid, EM = electron microscopy, AFM = atomic force microscopy, DF = dark field microscopy, HC = histochemistry (Warthin and Starry, Bosma-Steiner silver stain for spirochetes), IHC = immunohistochemistry, ISH = *in situ *hybridization, PCR = polymerase chain reaction, Bb = *Borrelia burgdorferi*, Wbl = Western blot, P = exact value of the significance calculated by the Fischer test, OR = Odds Ratio, CI = 95% confidence interval, + = positive, - = negative, Nd = not done, seq = sequence analysis, * = cases with mild or moderate AD-type cortical changes, ** = controls without any AD-type changes, *** = cases from previous studies, which were subtracted when the total number of cases of studies were considered, " = the number of 0 positive control was changed to 1 in order to calculate the exact OR and CI values.

In all these studies, which detected spirochetes using neutral techniques, 680 brain and blood samples were analyzed. In AD, more than 91.1% (451/495) of the samples were positive, while the 185 control samples were all negative.

### Periodontal pathogen spirochetes

Oral anaerobic Treponema (T) spirochetes are predominant periodontal pathogens, which are highly prevalent in the population. Several of them revealed to be invasive *in vivo *and *in vitro *[94,95]. Six different periodontal pathogen spirochetes, specifically, *T. denticola*, *T. pectinovorum, T. vincenti, T. amylovorum, T. maltophilum, T. medium *and *T. socranskii *were detected in the brains of AD patients using species specific PCR. At least one oral Treponema species was detected in 14 of 16 AD cases, and in 4 of 18 controls [96]. Species-specific antigens of *T. pectinovorum *and *T. socranskii *were observed in 15 AD and in 7 controls (P < 0.001). Six different Treponema species were detected in the brain in one AD patient, five species in four, four or three species each in one, and one species in seven AD cases. Of the four controls with Treponema spirochetes, one had two Treponema species and three one species each. The number of diverse Treponema species was significantly higher in the brains of AD patients compared to controls [96]. Treponema antigens were detected both in the hippocampus and frontal cortex. These important results, as proposed earlier [70,80-82], indicate that periodontal pathogen spirochetes in an identical way to *T. pallidum *have the ability to invade the brain, persist in the brain and cause dementia. They also indicate that co-infection by several spirochetes occurs in AD. These findings are in agreement with recent observations showing an association between periodontal diseases and AD [97].

### Borrelia burgdorferi

*B. burgdorferi *was first cultivated from the brain in two AD patients by MacDoald and Miranda [98] and MacDonald [99] and in 3 definite AD cases by Miklossy [85] (Table 1). Extensive characterization of the cultivated spirochetes confirmed, that the morphological, histochemical and immunohistochemical properties of these spirochetes are identical to those of *B. burgdorferi *[85,86]. Electron microscopic analysis demonstrated that they possess 10-15 endoflagella representative of *B. burgdorferi *species. 16S rRNA gene sequence analysis definitely identified the cultivated spirochetes as Borrelia species* sensu stricto *(*s. s.*) [85]. In two of these AD cases post mortem serological analyses of blood and cerebrospinal fluid (CSF) have revealed a positive serology for *B. burgdorferi *fulfilling the diagnostic criteria of the Center for Disease Control (CDC). *B. burgdorferi *specific antigens and genes were detected in the brains of these three AD patients where *B. burgdorferi *was cultivated. Neurofibrillary tangles were also immunoreactive with specific anti-*B. burgdorferi *antibodies and Borrelia antigens were co-localized with Aβ. Using *in situ *hybridization (ISH) *B. burgdorferi *specific OspA and flagellin genes were detected in senile plaques and in a number of neurofibrillary tangles [85]. Importantly, the cortical distribution of spirochete masses or colonies was identical to that of senile plaques. The pathological changes observed in the brain were similar to those occurring in the atrophic form of general paresis and in AD.

*B. burgdorferi *specific antigens were observed in the brain in an additional AD patient with concurrent Lyme neuroborreliosis [70]. Using species-specific PCR, *B. burgdorferi *DNA was detected in the brains in 5 of 16 AD patients and in one of 18 controls [96]. In these 6 positive cases (5 AD and 1 control) *B. burgdorferi *co-infected with oral Treponema spirochetes. *B. burgdorferi *specific DNA was detected by PCR in the brain of an additional patient with concurrent AD and Lyme neuroborreliosis [100] and in the hippocampus in 7 of 10 pathologically confirmed definite AD cases using PCR or ISH [101,102] (Table 1).

Pappolla et al., [103] who failed to detect *B. burgdorferi *in the brains of 6 AD cases and 4 controls concluded that the possibility of a different spirochete in AD not detectable by their methods could not be excluded, considering the possibility that several types of spirochetes may be involved in AD. Indeed, the goal of initial studies was not to show the involvement of *B. burgdorferi *alone in AD but that of the involvement of various types of spirochetes of the order *Spirochaetales*, including *B. burgdorferi*, oral, intestinal and other, yet uncharacterized spirochetes [70,80-84,86]. The title of the initial report, "Alzheimer's disease - A spirochetosis?", clearly indicates this goal [70].

In the two other studies where *B burgdorferi *was not detected in the brain, evidence is lacking whether the analyzed AD patients suffered from Lyme neuroborrelisosis [104,105] (Table 2). We cannot expect to detect *B. burgdorferi *in the brains of AD patients who have no Lyme neuroborreliosis. An example is the analysis of the involvement of *T. pallidum *in syphilitic dementia. If we would like to demonstrate the involvement of *T. pallidum *in dementia in a population without syphilis, we cannot succeed, despite the established fact that this spirochete can cause dementia. In order to study the involvement of *B. burgdorferi *in AD, the analysis of AD patients suffering from Lyme disease is necessary.

**Table 2:** Serological analysis of *Borrelia burgdorferi *in Alzheimer disease

Authors	N	Mat	Meth	AD	CTRL	Cult
Pappolla et al., 1989 [103]	47	CSF	ELISA	2/16	2/31	Nd

Gutacker et al., 1998 [104]	27	Bl,	ELISA, Wbl	1/27		Nd

Miklossy, 1993 and Miklossy et al., 2004 [70,85]	7	Bl, CSF	ELISA, IFAT, Wbl	2/4	0/3	+

Miklossy, 1993 [70]	1	Bl	ELISA	1/1		Nd

Meer-Scherrer et al., 2006[100]	1	Bl	ELISA, Wbl	1/1		Nd

MacDonald, 2006 [101,102]	1	Bl	ELISA, Wbl	1/1		Nd

Galbussera et al., 2008 [106]	98	Bl	ELFA	1(+/-)/50	0/48	Nd

Total: *Borrelia burgdorferi *serology

Blood:N = 135, AD: 7/84 (8.3%), CTRL: 0/51, (0%) "P = 0.2581, OR = 4.5, CI = 0.5-208

Blood & CSFN = 182, AD: 9/100 (9%), CTRL: 2/82 (2.43%), P = 0.1147, OR = 3.95, CI = 0.78-38

Data reviewed in the literature on the serological detection of *B. burgdorferi *specific antibodies in Alzheimer's disease. There is no statistically significant difference between AD and controls. The frequency of positive *B. burgdorferi *serology is more than eight times higher in the blood (7/84 versus 0/51) and almost four times higher when taken together blood and CSF (4.3% versus 2.5%) in AD compared to controls. The Odds ratio values (4.5 and 3.95) indicate that positive *B. burgdorferi *serology represents a risk for AD. N = total number of cases investigated, AD = Alzheimer disease, CTRL = control, Mat = material, Meth = methods, Cult = culture, AD = Number of AD cases with positive serology to *B. burgdorferi */number of AD cases analyzed, CTRL = Number of AD cases with positive serology to *B. burgdorferi */number of control cases analyzed, Bl = blood, CSF = cerebrospinal fluid, Wbl = Western blot, IFAT = Indirect Immunofluorescent Antibody Test, ELISA = Enzyme-Linked Immunoabsorbent Assay, ELFA = Enzyme-Linked Fluorescence Assay, P = exact value of the significance calculated by the Fischer test, OR = Odds Ratio, CI = 95% confidence interval, + = positive, Nd = not done, " = the number of 0 positive control was changed to 1 in order to calculate the exact OR and CI values.

Similarly, due to the low incidence of Lyme dementia compared to AD, the analysis of the seroprevalence of *B. burgdorferi *alone may be disappointing [104,106] (Table 2). In such studies it is difficult to prove the involvement of *B. burgdorferi *in AD and we cannot exclude the involvement of other spirochetes. As one may expect, there is no statistically significant difference between the positive blood and/or CSF serology between such AD and control populations (P = 0.1147). However, it is noteworthy, that the frequency of positive blood serology for *B. burgdorferi *is about 8 times higher in AD and considering both blood and CSF serology about 4 times higher (9%) compared to controls (2.43%). The high OR values (4.5 and 3.95, respectively) are also indicative of a higher risk of positive *B. burgdorferi *serology in AD. This is in harmony with the findings that in a statistically significant proportion of the AD population analyzed (25.3%) *B. burgdorferi *was detected in the brain. It is also noticeable that in all those studies, which show the involvement of *B. burgdorferi *in AD, the patients had a positive serology for *B. burgdorferi *and/or this spirochete was cultivated from the brain in BSK medium [70,85,98,99] or species-specific DNA was detected in the brain [96] indicating that these AD patients suffered from Lyme neuroborreliosis. Importantly, the majority of AD patients analyzed in these studies came from endemic areas of Lyme disease [70,85,98,99,101,102]. To consider, that the results may also vary depending whether the patients analyzed were living in endemic areas of Lyme disease is also important, when analyzing *B. burgdorferi*.

In future studies, to consider that several types of spirochetes can co-infect in AD [96] and that spirochetes frequently exhibit pleomorphism in host tissues [89] is also essential. In view of an infectious origin of AD the use of appropriate healthy control population without AD-type cortical changes and without other neuro-psychiatric disorders is also essential.

Taken together, these observations derived from various laboratories show that several types of spirochetes can infect the brain in AD and co-infection with several types of spirochetes occurs. As expected, the frequency of periodontal pathogen spirochetes is higher compared to that of *B. burgdorferi*, which is present in less then one third of the AD cases analyzed. The significantly higher frequency of *B. burgdorferi *in the brain of AD patients, the high risk factor and the results of the multifaceted analysis in three AD patients with concurrent Lyme neuroborreliosis, where *B. burgdorferi *was cultivated from the brain and species specific antigens and DNA were present in the cerebral cortex show that *B. burgdorferi *is involved in the pathogenesis of a subset of AD cases [85].

## Analysis of the association of spirochetes and AD

Based on the substantial data available in the literature, contingency tables were used to analyze the strength of the association between spirochetes and AD. Fisher test was used to assess whether the difference between the occurrence of spirochetes in AD and controls is statistically significant. Odds ratio (OR) and 95% confidence interval (CI) values were also computed. If in the control group the number of positive cases was 0 in order to calculate OR and 95% CI 1 positive control case was added (Table 1).

**Figure 1:**
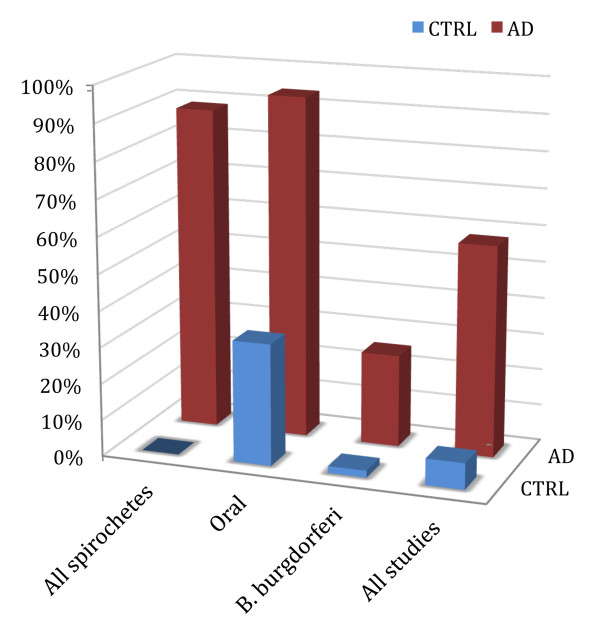
**Association of spirochetes with Alzheimer's disease**. The frequency of spirochetes is significantly higher in the brains of Alzheimer patients compared to controls. The statistical analysis is based on the cumulative data of the literature entered in Table 1. The association is statistically significant in the four groups analyzed: in the group where all types of spirochetes were detected using neutral techniques (All spirochetes), in the group of oral periodontal pathogen spirochetes (Oral spirochetes), in the group where *Borrelia burgdorferi *was detected alone (*B. burgdorferi*) and in the group where all studies were considered (All studies)

In those studies where all types of spirochetes were detected employing neutral techniques (Table 1, Figure 1), spirochetes were observed in the brain in 90.1% (64/71) of AD cases and were absent in controls without any AD-type changes (Table 1). The difference was significant (P = 4.8. × 10^-18^; OR = 274, 95% CI = 32-11345, N = 102). When cases with mild or moderate AD-type changes were also included as preclinical stages of AD, 91.5% of the cases (76/83) were positive (P = 1 × 10^-19^; OR = 325, 95% CI = 38-13440, N = 114). The difference remains significant when those cases were also included where spirochetes were analyzed in the blood (P = 1.1 × 10^-15^, OR = 105, 95% CI = 13-4329).

**Figure 2:**
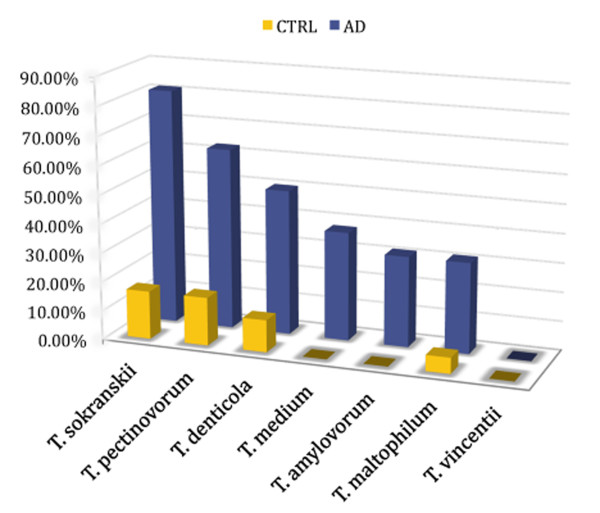
**Association of oral invasive periodontal Treponema (T.) spirochetes with Alzheimer's disease**. Using species specific PCR and antibodies six of seven periodontal pathogen spirochetes analyzed were detected in the brains of AD patients [96], (Table 1). The association of oral Treponemas with Alzheimer's disease is statistically significant [96], (Table 1).

The association between periodontal pathogen spirochetes and AD was statistically significant as well (Table 1, Figure 2). They were detected in the brain in 93.7% of AD and in 33.3% of control cases (P = 3.6 × 10^-4^; OR = 30; 95% CI = 2.8-1364; N = 34).

*B. burgdorferi *(Table 1, Figure 1) was observed 13 times more frequently in the brain in AD (19/75, 25.3%) compared to controls (1/52, 1.9%) (P = 2.9 × 10^**-4**^, OR = 17; 95% CI: 2 - 732; N = 127). The low prevalence of Lyme disease compared to AD is well reflected by the lower frequency (25.3%) of *B. burgdorferi *compared to the higher, more than 90% frequency of all types of spirochetes detected with neutral techniques or the highly prevalent periodontal pathogen spirochetes.

When considering all studies (Table 1, Figure 1) detecting all types of spirochetes and their specific species, their frequency was 8 times higher in the brain in AD (90/131 = 68.7%) compared to controls (6/71 = 8.45%). The difference is statistically significant (P = 1.7 × 10^-17^; OR = 23; 95% CI = 9-71, N = 202). The association remains strongly significant when the 12 cases with mild AD-type changes (P = 1.5 × 10^-19^, OR = 26, 95% CI = 10-80, N = 214) or those cases where spirochetes were analyzed in the blood were also included (P = 1.5 × 10^-17^, OR = 20, 95% CI = 8-60, N = 247). If considering errors, which may arise from those studies where the detection of spirochetes was restricted to *B. burgdorferi *alone, without considering other spirochetes, the percentage of spirochetes in AD would be even higher than 68.7%. This is supported by the high percentage of spirochetes in studies where all types of spirochetes were detected using neutral techniques (90.1%) or where the highly prevalent periodontal pathogen spirochetes were analyzed (93.7%).

Taken together, these results show a strong, statistically significant association between spirochetes and AD and show that these microorganisms represent a strong risk for AD.

## Further experimental evidence for a causal relationship between spirochetes and AD

Additional studies have brought further evidence in support of a probable causal relationship between spirochetes and AD. For these experimental studies *B. burgdorferi *was employed, as this spirochete can be cultivated in synthetic medium and maintained in pure culture.

When primary neuronal and glial cells and brain cell aggregates were exposed to *B. burgdorferi sensu stricto *spirochetes (*B. burgdorferi *strain B31 and strains ADB1 and ADB2 cultivated from the brains of AD patients), Thioflavin S positive and Aβ-immunoreactive "plaques" as well as tangle- and granular lesions similar to granulovacuolar degeneration were induced [107]. Spirochete induced Aβ accumulation was identified by Western blot and the β-pleated sheet conformation of the amyloid in spirochete-induced plaques was detected *in situ *using Synchrotron InfraRed MicroSpectroscopy (SIRMS). Borrelia induced tau phosphorylation and increased AβPP levels represented additional experimental evidences that spirochetes are able to induce an AD-type host reaction [107].

Both, reference Borrelia spirochetes (B31) and those cultivated from the brains of AD patients (ADB1 and ADB2 strains) invaded neurons and glial cells and induced nuclear fragmentation, indicating that these spirochetes are invasive [89,107]. They were located extra- and intracellularly. Their intracellular location indicates that they can be protected from destruction by the host immune reactions [70,89,107]. These results show that in an analogous way to *T. pallidum *they can persist in the brain and cause dementia, cortical atrophy and the pathological hallmarks of AD.

It is noteworthy that spirochetes frequently co-infect with other bacteria and viruses. Co-infection of *T. pallidum *with other bacteria, various Herpes viruses and *Candida albicans *was frequently observed in syphilis [91]. In Lyme disease, in addition to various co-infections transmitted by tick-bite (*e.g*. bartonellosis, ricketttsiosis, babesiosis etc.) *B. burgdorferi *frequently co-infects with other pathogens, which are independent of the tick-bite, *e.g*. *Clamydophyla pneumoniae *(*C. pneumonia*) [108] and Herpes viruses [108-111]. Co-infection of spirochetes with *C. pneumonia *also occurs in a Lyme-like tick-borne disease in Brazil [112]. Intriguingly, *C. pneumoniae *[113,114] and Herpes simplex type 1 (HSV-1) [115,116] were also detected in the brain in AD, suggesting that similarly to Lyme disease and syphilis, concurrent infection with several pathogens may frequently occur in AD as well.

*C. pneumoniae*, *H. pylori*, periodontal pathogens, including *T. denticola *and Herpes viruses are also linked to atherosclerosis [2,3,7], cardiovascular disorders [4,6,117] and diabetes mellitus [16,118,119], which indicate that these infectious agents, via hematogenous dissemination, may reach and infect various organs distant from the site of the primary infection. In agreement with this view, epidemiological studies revealed a close association between periodontal diseases and these chronic disorders [120]. It is noteworthy that AD is not only associated with these chronic inflammatory disorders but with chronic periodontal disorders as well [121].

## Mechanisms involved in spirochete-host interaction and their similarities to AD

The strong neurotropism of spirochetes is well known. Spirochetes can invade the brain and generate latent, persistent infection [29,63,65]. In addition to hematogenous dissemination, they can spread via the lymphatics and along nerve fiber tracts [63,91]. Accordingly, periodontal invasive spirochetes were detected along the trigeminal nerve and in trigeminal ganglia [96]. They might also propagate along the fila olfactoria and tractus olfactorius, which would be in harmony with the olfactory hypothesis [122-124] and with previous observations showing that the olfactory tract and bulb are affected in the earliest stages of the degenerative process in AD [125].

Spirochetes attach to host cells through their surface components, including collagen-binding proteins, bacterial amyloids and pore forming proteins [126-131]. Through activation of plasminogen and factor XII, bacterial amyloids contribute to inflammation and modulate blood coagulation [132].

The innate immune system enables host cells to recognize spirochetes, execute proinflammatory defenses, and start adaptive immune responses.

Pattern recognition receptors, located on the cell membrane of various cells, particularly on phagocytes and microglia recognize unique structures of spirochetes. The largest family of pattern recognition receptors is that of Toll-like receptors (TLRs). TLRs are also present in the brain [133]. Macrophages and microglia activated through TLR signaling secrete chemokines and cytokines and express various proinflammatory molecules for the removal of pathogens and affected cells. Spirochetes and their surface lipoproteins activate TLR signaling through CD14 [134,135]. As an example, tri- or di-acylated lipoproteins of *B. burgdorferi *bind to lipopolysaccharide binding protein (LBP), which activates TLR signaling through CD14 [136].

It is noteworthy, that in addition to spirochetal antigens and DNA, D-amino acids and bacterial peptidoglycan, two natural constituents of Prokaryotic cell wall unique to bacteria, were also detected in the brain in AD [83,84,137,138]. Pattern recognition receptors are upregulated in the brain in AD, and TLR2 and TLR4 gene polymorphisms influence the pathology of AD [139,140]. Activation of microglia with TLRs 2, 4 and 9 ligands markedly increases Aβ ingestion *in vitro *[141]. Finally, stimulation of the immune system through TLR9 in AβPP (Tg2576) transgenic mice results in reduction of Aβ deposits [142].

Once microorganisms are recognized, the activation of the innate immune system induces phagocytosis and bacteriolysis through the formation of the membrane attack complex (MAC, C5b9) [143-145] and promotes inflammatory responses. Activation of the clotting cascade generates bradykinin, which increases vascular permeability. Spirochetes activate both the classic and alternative pathways and induce acute phase proteins. Serum amyloid A (SAA) and C Reactive Protein (CRP) levels are elevated in *T. pallidum *and *B. burgdorferi *infections [146,147]. Through their ability to induce the production of tumor necrosis factor (TNF) by macrophages, spirochete lipoproteins play an important role in systemic and local inflammatory changes that characterize spirochetal infections [148].

In Alzheimer's disease, activated microglia that are designed to clean up bacteria and cellular debris surround senile plaques and extracellular neurofibrillary tangles [53]. Both the cellular and humoral components of the immune system reactions [48-53] and critical constituents of the classical and alternative complement pathways are associated with AD lesions [51,52,149].

Spirochetes are able to evade host defense mechanisms and establish latent and slowly progressive chronic infection. They employ a broad range of strategies to overcome antigenic recognition, phagocytosis and complement lysis. Blockade of the complement cascade allows their survival and proliferation even in immune competent hosts. Complement resistant strains of *B. burgdorferi *possess five Complement Regulatory Acquiring Surface Proteins (CRASPS), which bind to factor H (FH) and factor-H like protein-1 (FHL-1) of the alternative pathway [145,150]. Binding to the surface of spirochetes host FH and FHL-1 promotes the formation of inactive iC3b from C3b preventing MAC lysis. *B. burgdorferi *spirochetes possess a CD59-like complement inhibitory molecule as well [151], which by interacting with C8 and C9, inhibits binding of the opsonizing components C4b and C3b to MAC and consequently, prevents bacteriolysis [150]. Impaired complement lysis was also observed in *T. pallidum *infection [143].

*B. burgdorferi *protects itself from destruction by the host adaptive immune system as well. It induces interleukin-12 (IL-12), a cytokine critical for driving cellular responses toward Th1 subset [152-154]. This shift retards antibody production by Th2 cells against the spirochete. Intracellular survival of spirochetes also confers protection against destruction by the host defense reactions. Evasion of spirochetes will result in their survival and proliferation in the brain. Their accumulation in the cerebral cortex will lead to the formation of senile plaques, tangles and granulovacuolar-like degeneration as shown by historic observations in syphilis [61,62] and by current observations and *in vitro *experiments reviewed here (Fig. 7).

Accumulation in the brain of "paralytic iron" is characteristic in general paresis [59]. Free iron abolishes the bactericidal effects of serum and strongly enhances bacterial virulence [155-157]. It is necessary for bacterial growth and plays a pivotal role in infection and inflammation [155-157]. Iron increases the formation of reactive oxygen intermediates causing lipid peroxidation and subsequent oxidative damage of proteins and nucleic acids [155-157]. Iron, also accumulates in the brain in AD [155,158-160].

The production of reactive oxygen and nitrogen intermediates by innate immune cells is an effective host-defense mechanism against microbial pathogens. Activation of macrophages and other host cells by bacteria or LPS, including spirochetes and their lipoproteins generates substantial amount of nitric oxide (NO) [157], which is critical in bacterial clearance [161]. Nitric oxide also plays a central role in AD [162].

Chronic bacterial infections (*e.g. *rheumatoid arthritis, leprosy, tuberculosis, syphilis, osteomyelitis) are frequently associated with amyloid deposition. Based on previous observations we have suggested that amyloidogenic proteins might be an integral part of spirochetes and could contribute to Aβ deposition in AD [70]. Recent observations indeed showed that the BH (9-10) peptide of a beta-hairpin segment of *B. burgdorferi *outer surface protein A (OspA) forms amyloid fibrils *in vitro, *similar to human amyloidosis [163,164]. Recent observations also show that amyloid proteins constitute a previously overlooked integral part of the cellular envelope of many bacteria [163-168]. Bacterial amyloids have important biological functions and contribute to bacterial virulence and invasion of host cells [165,166].

Genetic mutations occurring in AD (AβPP, Presenilin 1 and 2) are related to the processing of AβPP and result in increased production of Aβ 1-42 and Aβ 1-43 [169]. AβPP revealed to be a proteoglycan core protein [170] and is involved in the regulation of immune system responses and in T cell differentiation [171-173]. Recent observations showed that Aβ is an innate immune molecule and belongs to the family of antimicrobial peptides AMPs [174], which are involved in innate immune responses. Consequently, genetic defects in AβPP, PS-I and PS-II should be associated with an increased susceptibility to infection. ApoE4, an important risk factor for AD, is also risk factor for infection and enhances increased expression of inflammatory mediators [175,176].

Promoter polymorphisms in pro-inflammatory cytokine genes facilitate infections [177]. TNF-α plays a critical role in host defenses against infection [178,179]. The influence of TNF-α on *T. pallidum *and *B. burgdorferi *infections has been repeatedly reported [153,180]. Human Leukocyte Antigen (HLA) gene polymorphism is a dominant marker of susceptibility to infection, including *B. burgdorferi *infection [181]. TNF-α and HLA polymorphisms, which are risk factors for infection, substantially influence the risk of AD as well [182-184].

## Analysis of causal relationship between spirochetes and AD following Koch's and Hill's postulates

### Koch's postulates

Koch's postulates were proposed to establish causal relationship between pathogens and specific diseases [34]. Following Koch's postulates I and II, the microorganism should be isolated from the affected tissue and grown in pure culture. Regarding Koch's postulates III and IV, the cultured microorganism should cause disease when introduced into a healthy host and must be re-isolated and identified as being identical to the original causative agent.

Spirochetes were cultivated from the brains of AD patients in a modified Noguchi medium and maintained in culture for about 1 month [70]. *B. burgdorferi *was cultivated from the brains of 5 out of 8 AD patients who suffered from Lyme neuroborreliosis and was maintained and propagated in pure culture [70,85,89], which fulfills Koch's postulates I and II. With respect to Koch's postulates III and IV the defining pathological and biological hallmarks of AD were reproduced *in vitro *not only in primary mammalian neuronal and glial cell cultures but in CNS organotypic cultures as well, which aim to replace *in vivo *studies [107]. *B. burgdorferi *(strains B31, ADB1, ADB2) was also recovered in pure culture from infected cell cultures [89]. *In vivo *studies might bring further evidence with respect to Koch's postulates III and IV. Following Koch's postulates the causal relationship between *B. burgdorferi *and dementia is much stronger, than in the case of *T. pallidum, *which is known to cause dementia, but cannot be cultivated in pure culture.

Koch himself acknowledged that the application of his postulates to establish causality is sometimes difficult and suggested that his criteria should be used as guidelines [35]. Indeed, like *T. pallidum*, several other bacteria and viruses cannot be grown in pure culture and based on his criteria to establish causality in chronic disorders is limited. In order to address this question, new criteria were proposed by Hill [36].

A previous review [185], on the analysis of association of infectious agent with AD following Hill's criteria concluded that the "treatment of chronic infection may become an important part of AD prevention and therapy". With respect to spirochetes only part of the historical and new data were included in this study.

Therefore, based on the substantial data available on the detection of spirochetes in AD, we analyzed the probability of a causal relationship following Hill's nine criteria [36].

### Hill's postulates

#### 1. Strength of the association

In agreement with Honjo et al. [185], the statistical analysis shows a significant association between spirochetes and AD (Table 1).

#### 2. Consistency of the association

Following Hill, the consistency of the association demands whether the results were "repeatedly observed by different persons, in different places, circumstances and times?". In 14 studies [70,80-85,90,96,98-102] spirochetes were detected in AD. Various authors in diverse laboratories, in different countries, using different techniques have detected spirochetes in AD, fulfilling Hill's claim for the consistency of association. In three studies [103-105], which failed to show the involvement of *B. burgdorferi *in AD, evidence is lacking whether the AD patients had a positive serology for *B. burgdorferi*, as for this goal, the analysis of AD populations suffering from Lyme neuroborreliosis would be essential. As mentioned by Pappolla et al. [103], the possibility of the involvement of other spirochetes in AD cannot be excluded. In another study on the analysis of sero-prevalence of *B. burgdorferi *in AD, due to the low incidence of Lyme dementia compared to AD can explain the negative result [106].

#### 3. Specificity of the association

Spirochetes and spirochete specific antigens and DNA associated with lesions defining AD indicate the specificity of the association.

#### 4. Temporality of the association

The temporal relationship of the association, is "... a question which might be particularly relevant with diseases of slow development... Have they already contracted it before?" *T. pallidum *infection in the atrophic form of general paresis is a historical example of temporal relationship between spirochetal infection and slowly progressive dementia [29,63,65]. Spirochetes were detected in AD patients with early stages of plaque-, tangle- and curly fiber-formation [83,84] indicating that infection takes place long before the diagnosis of dementia is made [70].

#### 5. Biological gradient of the association

That spirochetes are able to form plaque-, tangle- and curly fiber-like lesions [70,85,107] and their number progressively increases in the brains of patients with mild, moderate [83,84], and severe AD-type changes [70,80-87] fulfill this condition.

#### 6. Plausibility of the association

*T. pallidum *in the atrophic form of general paresis causes dementia, brain atrophy and Aβ deposition similar to the pathological and biological hallmarks of AD [61,62,67,85]. That AD-type pathological changes were also induced *in vitro *by *B. burgdorferi *and were observed in the brains of patients with concurrent AD and Lyme neuroborreliosis indicate that chronic spirochetal infection can cause dementia.

#### 7. Coherence of the association

As proposed by Hill, the cause-and-effect interpretation of the data should not seriously conflict with the generally known facts of the natural history and biology of the disease [36]. That a slow acting unconventional infectious agent acquired at an early age and requiring decades to become active may be involved in AD was never discarded [186,187]. Fischer, Alzheimer and their colleagues discussed the possibility that microorganisms may play a role in the formation of senile plaques and described similarities in the clinical and/or pathological manifestations of Alzheimer disease and general paresis [32,33,58,59,67,86]. Chronic spirochetal infection can cause slowly progressive dementia, cortical atrophy, chronic inflammation and Aβ deposition, which are indistinguishable from those occurring in AD [29,61,62,67,85,86]. Spirochete-host interactions result in various immune responses, free radicals, apoptosis and amyloid deposition, which are typical of AD [86]. The genetic defects occurring in AD can facilitate infection as well [for a review see 86]. Spirochetal infections cause cerebral hypoperfusion [188-190], cerebrovascular lesions and severely disturbed cortical capillary network [29,191,192], which are also important factors in the pathogenesis of AD [193-199]. As in AD, mixed forms of dementia due to cortical atrophy and vascular lesions frequently occur in neurospirochetoses [29,63], further strengthening the coherence of the association. All these observations indicate that, the association is in harmony with the natural history and biology of AD.

#### 8. Experimental evidences

**Figure 3:**
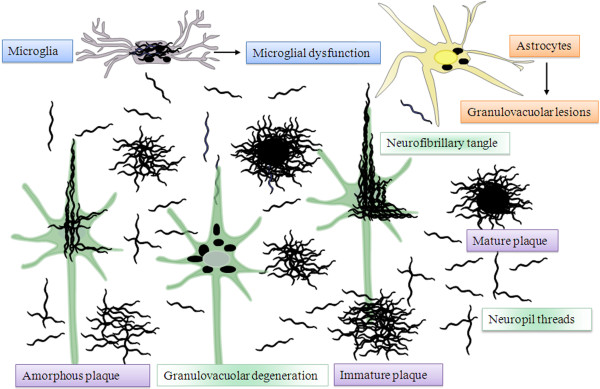
**Schematic representation of spirochetal invasion of the cerebral cortex reproducing the pathological hallmarks of Alzheimer's disease**. Spirochetes, in an analogous way to *Treponema pallidum, *form argyrophilic "plaques", colonies or masses along the cerebral cortex. Accumulation of spirochetes in masses reproduces the morphology of amorphous, immature and mature plaques. Agglutination of spirochetes in the center results in a homogeneous central core, which attract microglia. Spirochetes invading neurons lead to the formation of neurofibrillary tangles, and their pleomorphic granular form to granulovacuolar degeneration. Individual spirochetes disseminate along the cerebral cortex forming neuropil threads or curly fibers. Invasion of astrocytes by spirochetes can results in a similar granular pathology as in neurons. Spirochetes can also invade microglia, which may lead to their dysfunction and diminish their capacity to fight infection. Lesions similar to plaques, tangles and granulovacuolar degeneration were all reproduced by exposure of mammalian CNS cells and organotypic cultures to spirochetes [107].

Following exposure of primary mammalian neuronal and glial cells and brain organotypic cultures to spirochetes, lesions similar to the defining pathological and biological hallmarks of AD were produced [107] representing experimental evidence in favor of a causal relationship between AD and spirochetes. These experimental data [107,89] indicate that as observed in syphilis [29,61,62] and Lyme neuroborreliosis [85,89], the evasion of spirochetes can result in their survival and proliferation and the production of lesions similar to senile plaques, tangles and granulovacuolar-degenerations (Figure 3). Additional experimental data include transmission of Aβ amyloidosis to experimental animals [200-203], the observations showing the immune regulatory function of APP [171-173], the antimicrobial properties of Aβ [174] and the improvement in symptoms of AD patients following antibiotic treatment [204-208]. Further research and clinical trials would be primordial.

#### 9. Analogy of the association

The analogy of clinical and pathological hallmarks of AD to those of the atrophic form of general paresis and Lyme neuroborreliosis as revealed by historic observations and based on retrospective studies meets this condition [29,33,61,62,67,85,86].

Taken together, the analysis of historic and recent data available in the literature following Koch's and Hill's criteria is in favor of a causal relationship between neurospirochetosis and AD.

#### Conclusions

Various types of spirochetes, including *B. burgdorferi*, and six periodontal pathogen spirochetes (*T. socranskii*, *T. pectinovorum*, *T. denticola, T. medium, T. amylovorum *and *T. maltophilum*) were detected in the brains of AD patients. The pathological and biological hallmarks of AD, including increased AβPP level, Aβ deposition and tau phosphorylation were induced by spirochetes *in vitro*. The statistical analysis showed a significant association between spirochetes and AD. The strongly significant association, the high risk factor and the analysis of data following Koch's and Hill's criteria, are indicative of a causal relationship between neurospirochetoses and AD.

Spirochetes are able to escape destruction by the host immune reactions and establish chronic infection and sustained inflammation. I*n vivo *studies with long exposure times will be necessary to efficiently study the sequence of events and the cellular mechanisms involved in spirochete induced AD-type host reactions and Aβ-plaque, "tangle" and "granulovacuolar" formation. The characterization of all types of spirochetes and co-infecting bacteria and viruses is needed, in order to develop serological tests for the early detection of infection. The pathological process is thought to begin long before the diagnosis of dementia is made therefore, an appropriate targeted treatment should start early in order to prevent dementia.

Persisting spirochetal infection and their persisting toxic components can initiate and sustain chronic inflammatory processes through the activation of the innate and adaptive immune system involving various signaling pathways. In the affected brain the pathogens and their toxic components can be observed, along with host immunological responses. The response itself is characteristic of chronic inflammatory processes associated with the site of tissue damage. The outcome of infection is determined by the genetic predisposition of the patient, by the virulence and biology of the infecting agent and by various environmental factors, such as exercise, stress and nutrition.

The accumulated knowledge, the various views, and hypotheses proposed to explain the pathogenesis of AD form together a comprehensive entity when observed in the light of a persisting chronic inflammation and amyloid deposition initiated and sustained by chronic spirochetal infection. As suggested by Hill, once the probability of a causal relationship is established prompt action is needed. Similarly to syphilis, one may prevent and eradicate dementia in AD. The impact on healthcare costs and on the suffering of the patients would be substantial.

## Competing interests

The author declares that they have no competing interests.

## Authors' contributions

JM wrote the manuscript and approved the final version of the manuscript.
